# Management of adult patients with tinnitus: Preparedness, perspectives and practices of audiologists

**DOI:** 10.4102/sajcd.v66i1.621

**Published:** 2019-11-19

**Authors:** Firdaus Dawood, Nasim B. Khan, Vedika Bagwandin

**Affiliations:** 1 Discipline of Audiology, University of KwaZulu-Natal, Durban, South Africa

**Keywords:** Audiologists, speech therapist and audiologists, STA’s; tinnitus, intervention, management

## Abstract

**Background:**

Audiologists, globally, are generally challenged when assessing and creating intervention plans to help patients suffering from tinnitus. Tinnitus is very common among individuals and may significantly affect one’s quality of life, especially if not addressed by health care professionals. In South Africa, there seems to be limited published studies regarding the current practices of tinnitus management by audiologists. This is mainly because of limited training and a lack of guidelines and strategies for the management of tinnitus. In particular, some participants reported being unfamiliar on how to approach the identification of tinnitus and difficulty is also encountered when counselling tinnitus patients.

**Aim:**

The aim of this study was to describe the preparedness, perspectives and practices of audiologists who manage adult patients with tinnitus.

**Method:**

Two hundred and forty-three registered Health Professions Council of South Africa (HPCSA) participants were involved in the study by responding to an electronic questionnaire survey. Data were collected online from Survey Monkey and were exported to Statistical Packages for the Social Sciences (SPSS) (Version 23) for statistical analysis. Data were analysed using descriptive and inferential statistics. Closed-ended questions were analysed within a quantitative framework and thematic analysis for open-ended questions that were descriptively quantified.

**Results:**

The results of the study are presented according to the objectives. Approximately 44% of participants (44.3%) disagreed that the undergraduate university training had sufficiently prepared them to manage adult patients with tinnitus. Very few (12.3%) had the opportunity to attend specialist training on how to assess patients with tinnitus. Similarly, only 11.6% received any specialist training with regard to tinnitus intervention. With regard to its overall management, 49.4% felt adequately informed in the assessment of patients with tinnitus, while a further 39.2% rated their experience as being limited with regard to tinnitus intervention. There is no statistical significance relationship between participants’ years of experience and tinnitus intervention (*p* = 0.075). Most participants did not follow any standard guidelines for its management. Some participants (26.8%) reported that further education and training are required in the overall management of patients with tinnitus, while a further 17.7% required training in all areas of tinnitus.

**Conclusion:**

The feedback relating to the study suggests that overall management of tinnitus seems to be a challenge among South African audiologists, irrespective of their years of experience. Audiologists in the study perceived that tinnitus services are limited mainly because of a lack of or limited knowledge, training and guidelines, these being affected by contextual restraints.

## Introduction

Tinnitus is commonly understood as the sensation or perception of sound that a person experiences mainly in the absence of external auditory stimuli (Langguth, Kreuzer, Kleinjung, & De Ridder, 2013). There are reports of underlying clinical causes associated with tinnitus that include middle ear and cardiovascular diseases, nasal allergies, autoimmunity, diabetes, degenerative neural disorders and socio-demographic and environmental factors (Heller, 2003; Hoffman & Reed, 2004; Sindhusake et al., 2003; Vernon & Meikle, 1998 all as cited in Manche, Madhavi, Meganadh, & Jyothy, 2016). The aetiology of tinnitus is not yet known or fully understood, and can arise from pathological changes within the auditory system (Langguth et al., 2013), its pathophysiology being considered a controversial area in the field of medical science (Swain, Nayak, Ravan, & Sahu, 2016). There are variations in the prevalence of tinnitus among epidemiological studies globally (Henry & Wilson, 2001). Approximately 10% – 15% of adults experience tinnitus, of whom approximately 20% require clinical intervention (Davis & Refaie, 2000; Hoffman & Reed, 2004; Jastreboff & Hazell, 2004 all as cited in Henry, Zaugg, Myers, & Schechter, 2008). There is a strong association between hearing loss and tinnitus, which has resulted in many audiological associations and regulatory bodies noting tinnitus as a significant component of audiological practice (Moller, Langguth, De Ridder, & Kleinjung, 2011).

Approximately 20% of individuals experience tinnitus to the extent that their quality of life, well-being and productivity are diminished (Jastreboff & Hazell, 2004; Sullivan et al., 1988). Numerous studies have reported on the adverse effects of tinnitus, which include insomnia, depression, impaired concentration and impaired quality of life (Moroe & Khoza-Shangase, 2014; Sourgen & Ross, 1998). Audiologists providing tinnitus services should, therefore, have knowledge on the extent to which patients are affected by tinnitus (Bagwandin & Joseph, 2017), highlighting the need for the overall management of tinnitus as patients require interventions to address this condition.

Audiologists are considered an essential part of the team to assist patients with tinnitus because of their extensive professional training and experiences (Henry, Schechter et al., 2005). Audiologists are trained regarding auditory disorders and functioning, acoustics and psychoacoustics, which are important for the overall management of tinnitus. However, there are variations in the audiology training programmes that are intended to prepare them for this role (Henry, Schechter et al., 2005). An audiological evaluation of tinnitus is essential, and includes facilitating the following measures: an otoscopic examination, tympanometry, pure tone threshold evaluation including high frequencies and interoctaves and speech tests (Henry, Schechter et al., 2005; Tunkel et al., 2014). A detailed case history is the key to measuring the disability or handicap that is associated with tinnitus (Sweetow et al., 2000). After an audiologic assessment, the basic evaluation of tinnitus should include but not be limited to the following measures: a comprehensive case history, psychoacoustic testing that includes most comfortable levels, loudness discomfort levels, tinnitus pitch matching with octave confusion, loudness matching minimum masking levels and residual inhibition as well as the use of subjective questionnaires (Sweetow et al., 2000).

The type of management implemented for tinnitus patients is determined by the experience of clinicians (Hall, Szczepek, Kennedy, & Haider, 2015). Emphasis is placed on the audiologists’ familiarity with hearing aids and their ability to reduce the effects of tinnitus by using auditory stimulation (Sheldrake & Jastreboff, 2004, as cited in Henry & Zaugg et al., 2005). Furthermore, audiologists have counselling skills that are necessary to manage patients’ symptoms, facilitate a better understanding of their condition and help patients achieve a form of acceptance to cope (Hall & Ruth, 1999). The belief and attitude of patients towards tinnitus may play a significant role in their rehabilitation process (Erlandsson et al., 1992 as cited in Sourgen & Ross, 1998), with patients needing to be given the necessary information to live with or overcome the condition (Tyler & Babin, 1993 as cited in Sourgen & Ross, 1998).

To date, there are no international standards to manage tinnitus, with research being the best avenue to ensure that better practices are available for good management (Tyler, 1999). Tinnitus management strategies are diverse among patients, being based on their various aetiologies and characteristics (Powers & Ramirez, 2014). Tinnitus retraining therapy and cognitive behavioural therapy are the most documented methods of management to assist patients with tinnitus (Langguth et al., 2013), and more research in this area would ensure for better practice as there is no international standard being implemented for the overall management of tinnitus. It has been reported that in the United States, for example, the practice of tinnitus management challenges health care professionals as graduate programmes differ with regard to the degree and type of training provided (Henry, Dennis et al., 2005). A local study highlighted the need for audiologists to be adequately trained to assist patients with tinnitus and for a more uniform approach to be in place (Moroe & Khoza-Shangase, 2014). As a result, audiologists may not be adequately trained on how to manage it, which adds to the challenges they face in advising their patients. Audiologists therefore need to be proactive by keeping updated with the latest literature in this field and attending continuous professional development workshops on tinnitus. This may help them keep informed and be open to new procedures to effectively implement suitable strategies to meet the needs of their patients.

A study in South Africa conducted by Naidoo (2006) reported an absence of published data regarding implemented tinnitus management approaches and interventions. It was further recommended that the development of guidelines on the management of tinnitus in South Africa should address the diverse practices of tinnitus management among audiologists. This may also encourage health care professionals with limited undergraduate training to practise confidently in this area (Naidoo, 2006). The availability of practice guidelines will assist in reducing the variations in clinical care (Tunkel et al., 2014**)**, and will ensure that effective tinnitus services are provided, specifically in South Africa, where no standards are currently in place.

A study conducted in South Africa revealed that limited tinnitus services were provided to adult patients because of minimal and varied training of audiologists in the area (Naidoo, 2006). The main reason for audiologists not practising tinnitus management in both the public (46.15%, *n* = 12) and private sector (41.17%, *n* = 7) were because of insufficient training among health care professionals. However, the necessity for tinnitus management was recognised in the study, and the management of tinnitus was practised routinely more in the private (23.29%) than public (12.5%) sectors (Naidoo, 2006). In the United Kingdom, it was reported that limited time is a factor when interacting with tinnitus patients (Hoare, Gander, Collins, Smith, & Hall, 2010). Schechter and Henry (2002) have acknowledged that because of the fast-pace nature of audiology clinics, longer time slots for tinnitus care may not be possible at all institutes; however, this may work in the private sector. Another patient perspective study revealed that 21 (78%) of 27 participants reported that coping strategies were not provided to them, while nine (33%) implemented their own behavioural coping strategies (Moroe & Khoza-Shangase, 2014). The study findings may be interpreted with caution as a smaller sample size was used with an unequal gender distribution, which limited the generalisability to the general tinnitus-suffering population (Moroe & Khoza-Shangase, 2014). A study by Rau (2004 as cited in Moroe & Khoza-Shangase, 2014) concluded that audiologists in private practice did not feel adequately trained and were not familiar with techniques to treat tinnitus because of insufficient training in this area.

In view of the complex nature of tinnitus, its assessment is a challenge as patients often receive different levels of service from audiologists, its management generally not being part of all audiological training programmes (Schechter & Henry, 2002). The use of questionnaires can be beneficial to determine the impact of tinnitus (Meikle et al., 2012) and to develop intervention plans that are patient specific (McCombe et al., 2008). The Tinnitus Handicap Inventory, Tinnitus Handicap Questionnaire, Tinnitus Reactions Questionnaire and the Tinnitus Questionnaire have been selected unanimously by professionals with experience in tinnitus to use when determining the severity of tinnitus in patients (Hall et al., 2015). However, Meikle et al. (2012) discuss the Tinnitus Functional Index, which documents the validity of determining the impact of the severity of the tinnitus. In terms of intervention, studies have documented efficacy with the following techniques: sound, habituation, tinnitus retraining and cognitive behavioural therapy (De Barros Suzuki, Suzuki, Yonamine, Onishi, & Penido, 2016; Hobson, Chisholm, & El Refaie, 2012; Falkenberg & Wie, 2012; Andersson, 2002). Audiologists may have counselling skills that are necessary to manage the symptoms, which may facilitate a better understanding of tinnitus by the patient and learn strategies to cope with tinnitus (Hall & Ruth, 1999). Counselling by audiologists can help the patient to understand the impact of their tinnitus, while education and management options may reduce any fear or anxiety experienced by the patient and alleviate the severity of their condition (Keaton, 2014).

Limited studies relating to tinnitus have been conducted in South Africa, some of which noted the need for appropriate tinnitus services to enable patients to lead a better quality of life (Moroe & Khoza-Shangase, 2014; Naidoo, 2006, Sourgen & Ross, 1998). However, the measures taken by audiologists to manage patients with tinnitus remain unclear (Naidoo, 2006). The limited provision of services and poor management strategies may be because of a number of reasons, the most apparent being the absence of established standards to support audiologists and inadequate training, which results in a lack of knowledge and confidence. Furthermore, studies have primarily focused on the impact that tinnitus has on the daily activities in the adult population (Moroe & Khoza-Shangase, 2014; Sourgen & Ross, 1998), and there seems to be a lack of literature relating to the services provided by audiologists to patients with tinnitus.

## Method

### Objectives

The study had the following objectives:

- to describe audiologists’ preparedness regarding the overall management of tinnitus in adult patients
- to describe audiologists’ perspectives and practices relating to assessing and providing interventions for adult patients with tinnitus
- to obtain insight regarding the challenges and limitations hindering the overall management of adult patients with tinnitus and make recommendations for the improvement thereof.

### Research design

A non-experimental survey research design was used to obtain quantitative data from a questionnaire survey completed by speech therapists and audiologists that was completed online. In 2016, 1822 audiologists and speech therapists and audiologists were registered with the Health Professions Council of South Africa (HPCSA, 2016) were contacted to participate in the study. Of these, 243 (13 %) participated in the online survey, with nine incomplete questionnaires being excluded, their details being provided in Table 1.

**TABLE 1:** Biographical details (*n* = 243).

Characteristic	Values	*N*	%
Gender	Male	34	14
	Female	209	86
Years’ experience	<1	33	13.6
	1–5	100	41.2
	6–10	67	27.6
	>10	43	17.1
Employment location	Urban	176	72.4
	Rural	67	27.6
Domain of current employment	**Public health – DOH:**	-	-
	Hospital or clinic	115	45.6
	School	7	2.8
	**Private health:**	-	-
	Hospital or clinic	96	38.1
	School	3	1.2
	Academic	12	4.8
	Not in practice	12	4.8
Registration	Audiologist	131	53.9
	Speech Therapist and Audiologist	112	46.1
Highest qualification	B. Audiology	110	45.3
	B. STA	95	39.1
	Master	36	14.8
	Doctorate	2	0.8
Province	Eastern Cape	11	4.4
	Free State	4	1.6
	Gauteng	98	38.9
	KwaZulu-Natal	66	26.2
	Limpopo	20	7.9
	Mpumalanga	7	2.8
	Northern Cape	4	1.6
	North West	6	2.4
	Western Cape	27	10.7

B, Bachelor of; DOH, Department of Health; STA, speech therapist and audiologist.

### Research procedure

Prior to data collection, the HPCSA was informed about the research aims and procedures, and permission to access the postal addresses of the registered audiologists was obtained. All registered professionals were invited to participate in the study through an online portal, Survey Monkey. Participants were informed about the purpose of the study, the ethical considerations that would apply and were required to confirm their willingness to participate. Audiologists registered with the HPCSA and have worked with adult patients with tinnitus were part of the inclusion criteria. The information letter, consent form and questionnaire were uploaded onto Survey Monkey. The devised questionnaire was based upon studies by Hoare et al. (2010) and Hoare, Broomhead, Stockdale and Kennedy (2015), which was used as a guide and adapted to suit the South African context. The questionnaire was divided into four sections and consisted of 33 tick box and two open-ended questions, which took 10–15 min to complete. The sections were biographical details, preparedness to manage tinnitus, views and practices of audiologists regarding assessment and interventions, challenges experienced and recommendations.

An extensive literature review was done, and the current survey devised for this study was based upon research studies by Hoare et al. (2010) and (2015) and ensured it was suitable to the context of practising audiologist and/or speeach therapist and audiologist. In addition, a qualified audiologist with expertise in the area of tinnitus reviewed and validated the research tool. Five participants took part in the pilot study, with minor modifications being made. The electronic medium of collating responses via Survey Monkey was effective and maintained confidentiality, with no software issues. Data were collected online from Survey Monkey and exported to SPSS (Version 23) for statistical analysis. A descriptive statistical analysis of the data was initially conducted prior to conducting inferential analysis to assess associations between any categorical variables.

### Ethical consideration

All ethical principles that govern research were maintained throughout the study. The University of KwaZulu-Natal’s Humanities and Social Research Ethics Committee provided ethical clearance (HSS/0609/016M).

## Results

The results are presented with respect to the three objectives, with the demographic details being provided in Table 1.

The majority of participants (*n* = 209, 86%) were female, with less than 5 years’ experience and were based in urban areas. Approximately 85% had an undergraduate degree, with 14.8% of participants having a postgraduate degree, and worked in either a public or a private sector hospital or clinic. The majority were from Gauteng and KwaZulu-Natal provinces, which are the two most populous provinces in the country (Statistics South Africa, 2011).

### To describe the preparedness of participants for the overall management of tinnitus in adult patients

Some participants *disagreed* (*n* = 103, 42%) that they were sufficiently prepared for the overall management of tinnitus (*n* = 104, 43%) and reported being *unsure* regarding the availability of courses for tinnitus management in South Africa. Very few had undertaken specialist training to *assess* (*n* = 29, 12.3%) and *intervene* (*n* = 27, 11.6%) with patients with tinnitus. However, despite these participants indicating perceived lack of undergraduate training, only approximately one-quarter reported the need for further education and training in the *overall management of tinnitus* (*n*=139, 26.8%), while less than nine (1.7%) felt comfortable managing these patients. This may be because of participants not practising in the area of tinnitus and, therefore, did not see the need for further education and training in tinnitus (Table 2). Descriptive statistics analysed frequency responses and percentage calculations obtained from the closed-ended questions for this study.

**TABLE 2:** Further education and training of tinnitus.

Further education and training	*N*	%
Nothing, I am comfortable working with tinnitus	9	1.7
Assessment	94	18.1
Intervention	116	22.4
Overall management of tinnitus	139	26.8
Counselling	67	12.9
I require further education in all areas of tinnitus	92	17.7

### To describe the perspectives and practices of participants relating to the *assessment and intervention* of adult patients with tinnitus

The results indicated that half (*n* = 116, 49.4%) felt *adequately informed* to assess tinnitus in adult patients, while 100 (42.5%) felt *poorly or very poorly informed*, and only 19 (8.1%) were confident in their ability. Participants with *less than a year* of working experience indicated their knowledge of assessment was mostly *adequate* (*n* = 21), *poorly informed* (*n* = 7) and *very poorly informed* (*n* = 3) (Table 3). Participants with *more than 10 years* of working experience also indicated that they are *adequately* (*n* = 8) and *poorly informed* (*n* = 5), with some participants in this category also feeling *very poorly informed* (*n* = 2). Based on the results, there was no statistically significant relationship between the two variables (*p* = 0.394).

**TABLE 3:** Years of experience and extent of being informed about assessment.

Characteristic: age and extent of being informed about	*N*	%	Very poorly informed	Poorly informed	Adequate	Well informed	Very well informed
< 1	32	13.6	3	7	21	1	0
1–5	98	41.2	6	37	48	7	0
6–10	65	27.6	2	28	29	5	1
> 10	40	17.1	2	15	18	3	2

Risk factors for tinnitus that were most familiar by participants included noise exposure (*n* = 145, 34.6%), infections (*n* = 92, 21.9%) and head or neck injuries (*n* = 52, 12.4%), suggesting familiarity of factors that warrant further management. Other risk factors included hearing loss (presbycusis and otosclerosis), ototoxic medication (TB and HIV and AIDS), stress, anxiety, impacted cerumen, headaches and dizziness. Case history (*n* = 232, 22.1%), otoscopy (*n* = 210, 20%) and routine audiological testing (*n* = 215, 20.5%) were largely undertaken as part of the tinnitus assessment along with the use of tinnitus questionnaires (*n* = 140, 13.3%) (Table 4).

**TABLE 4:** Test battery used to assess tinnitus.

Test battery used to assess tinnitus	*N*	%
Case history	232	22.1
Otoscopy	210	20
Routine audiological testing	214	20.5
Tinnitus questionnaire	140	13.3
Loudness and pitch matching	117	11.1
Loudness discomfort levels	64	6.1
Minimum masking level	45	4.2
Residual inhibition	14	1.3
Do not conduct any	5	0.4
Other	6	0.5

Participants rated their professional experience as *limited* (*n* = 91, 39.2%) and *adequate* (*n* = 87, 37.5%) for the intervention of tinnitus in adult patients. With regard to the level of satisfaction experienced by participants when providing tinnitus services, it is observed that 78 (33.6%) participants felt neither satisfied nor dissatisfied. More participants leaned towards a negative response feeling *dissatisfied* (*n* = 73, 31.5%), while fewer participants felt *satisfied* (*n* = 57, 24.6%) with the provision of services offered. Participants with varied years of experiences reported their experience of working with tinnitus patients as *limited* (*n* = 11) and *very limited* (*n* = 10), respectively. The majority of participants with greater periods of working experience (i.e. >10 years) indicated their experience as *limited* (*n* = 13). There does not seem to be a statistically significant relationship between participants’ years of experience and tinnitus intervention (*p* = 0.075) (Table 5).

**TABLE 5:** Years of experience and intervention of tinnitus.

Characteristic: Years of experience and intervention of tinnitus	*N*	Very limited	Limited	Adequate	Good	Extensive
< 1	30	10	11	9	0	0
1–5	98	19	41	32	6	0
6–10	65	6	26	27	6	0
> 10	39	4	13	19	2	1

Most participants *did not follow* any standard procedures nor standard guidelines or policies to assess adult patients with tinnitus (*n* = 113, 48.1%) (*n* = 147, 59.5%), respectively. This question was targeted to determine whether there has been a change in this regard and in comparison to earlier studies. Participants responded positively (*n* = 142, 61.2% – *strongly agree*; *n* = 62, 26.7% – *agree*) for the development of guidelines to manage tinnitus. The most common guidelines used by participants for the assessment and intervention of tinnitus are presented in Table 6.

**TABLE 6:** Use of guidelines or policies for the assessment and intervention of tinnitus.

Use of guidelines or policies for assessment of tinnitus	*N*	%	Use of guidelines or policies for intervention of tinnitus	*N*	%
Good Practice Guide – NHS (2009)	25	10.1	Good Practice Guide – NHS (2009)	20	8.1
Clinical Practice Guide for Tinnitus – American Academy of Otolaryngology (2014)	30	12.2	Clinical Practice Guide for Tinnitus – American Academy of Otolaryngology (2014)	33	13.4
Clinical Guide of Tinnitus Management (ATM1)	25	10.2	Clinical Guide of Tinnitus Management (ATMII) – Management	21	8.3
Do not use any guide	147	59.5	Do not use any guide	157	63.6
Other†	20	8.1	Other†	16	6.5

† , Other included training materials from Oticon, Widex, Beltone and use of Jastreboff Tinnitus Retraining Therapy (TRT) model as well as Cognitive Behavioural Therapy (CBT).

Participants mostly implemented counselling techniques as part of the intervention process, followed by sound generators or tinnitus maskers, TRT and providing educational support or awareness. The data also indicated a combination of the above to aid in the provision of effective intervention services (Table 7).

**TABLE 7:** Techniques used by participants as part of their intervention with tinnitus patients.

Techniques used for tinnitus intervention	*N*	%
Sound generators or tinnitus maskers	150	23.6
Tinnitus retraining therapy	143	22.5
Educational support or awareness	120	18.9
Counselling	211	33.2
Other†	10	1.5

† , Other includes CBT, referrals for further medical investigations.

Participants in private practice (*n* = 39, 24%) seemed to create and implement more intervention plans for tinnitus in contrast than those in public health sector (*n* = 27, 10%). Most participants expressed an interest in developing a tinnitus management programme at their institute (*n* = 162, 69.8%), while 6.5% (*n* = 15) responded that this was not possible because of time constraints, understaffing issues and lack of interest in the area of tinnitus. With regard to referrals, ear, nose and throat specialists (ENTs) (*n* = 199, 36.3%) received most referrals from audiologists for further management of tinnitus patients.

### To understand the challenges and limitations hindering the overall management of adult patients with tinnitus and make recommendations for its improvements

Two open-ended questions were included for participants to highlight their challenges and provide recommendations to address these concerns. These responses were quantified, with the following key elements emerging (Tables 8 and 9). Participants reported a number of factors that challenged their ability to effectively assist in the overall management of tinnitus.

**TABLE 8:** Challenges regarding managing adult patients with tinnitus (*n* = 89).

Challenging areas	*N*	%	Some examples as illustrations of direct quote(s) and participants details
Assessing and managing tinnitus	47	53	‘I believe I have limited knowledge and experience in this field, everything is challenging from assessment to management’. (No. 102, Bachelor degree, 6–10 years’ experience, Private hospital/clinic) ‘I was not sufficiently trained in tinnitus, assessing and managing the patient is quite difficult’. (No. 160, Bachelor degree, 6–10 years’ experience, Public hospital/clinic)
Need for more counselling	35	39	‘Explaining the cause of tinnitus and counselling patients on coping with tinnitus especially since there is no quick fix medication that patients look for’. (No. 205, Bachelor degree, 1–5 years’ experience, Public hospital/clinic)
Language barriers	30	33.7	‘Language barrier is a huge problem as the questionnaires are only in English. Patients often do not see the importance of keeping a tinnitus diary so it makes rehabilitation difficult. Most patients want a quick fix for the problem’. (No. 213, Bachelor degree, 6–10 years’ experience, Public hospital/clinic)
Limited resources or funding	28	31.4	‘Lack of resources or equipment, include and enforce the urgency of the resources or equipment’s in the hospital budget plan’. (No. 68, Bachelor degree, 6–10 years’ experience, Public hospital/clinic) ‘Funds are limited within the public sector therefore we are not always capable of providing the best possible intervention to our patients. At this point in time, it is more important to buy hearing aids with the financial resources available than to purchase expensive tinnitus-specific hearing aids/resources’. (No. 249, Bachelor degree, <1 year’s experience, Public hospital/clinic)
Lack of protocols or guidelines	13	15	‘Guideline for assessment, including resources – management protocols that can be used as a benchmark to assist in improving and development of standards of care associated with tinnitus management’. (No. 156, Bachelor degree, <1 year’s experience, Public hospital/clinic)
Time constraints	7	8	‘Mostly lack of time and language barriers which hinder counselling process’. (No. 213, Bachelor degree, 6–10 years’ experience, Public hospital/clinic)
Lack of affordability to access services because of location	3	3.37	‘Shortage of space for movements during assessment, Geographical locations: PTS cannot afford to come back to the hospital for regular follow-up and management’. (No. 172, Bachelor degree, 6–10 years’ experience, Private hospital/clinic)
Cultural beliefs	2	2.2	‘Cultural beliefs on causes of tinnitus, retraining strategies may not be relevant to population in rural area, knowing the correct policy to follow and have confidence during assessment and management of such patients’. (No. 125, Bachelor degree, 6–10 years’ experience, Public hospital/clinic)

**TABLE 9:** Recommendations to improve the tinnitus management in adult patients (*n* = 100).

Recommendations	*N*	%	Some examples as illustrations of direct quote(s) and participants details
Further training to assess and manage	39	39	‘Not confident enough in the overall management of tinnitus. I do my level best with my patients, would like to do a whole lot more with further training on tinnitus’. (No. 96, Bachelor degree, 1–5 years’ experience, Public hospital/clinic)
Guidelines	32	32	‘More clear guidelines that are easily available and widely circulated’. (No. 169, Bachelor degree, <1 year’s experience, Public hospital/clinic)
Access to information or further research	30	30	‘Dearth of information. Patients reluctance to continue with management’. (No. 135, Master’s degree, 10 years’ experience, Private hospital/clinic) ‘More elaborate information on tinnitus, which includes the identification, intervention, diagnosis, and management of tinnitus’. (No. 108, Bachelor’s degree, <1 year’s experience, Not in practice)
Undergraduate training	25	25	‘Audiology undergraduate training programme needs to provide adequate training in tinnitus assessment and intervention’. (No. 234, Bachelor degree, 1–5 years’ experience, Public school)
Courses or workshops for tinnitus	21	21	‘I see tinnitus patients frequently and I think more workshops should be created to assist us in managing these patients’. (No. 56, Bachelor degree, 1–5 years’ experience, Private hospital/clinic)
Awareness or education to health care professionals	30	30	‘Essentially, it is up to the professional and more personal effort should be made theoretically and clinically’. (No. 188, Bachelor degree, 1–5 years’ experience, Public hospital/clinic) ‘Awareness of what audiologists are able to do to help … Adherence to treatment plan from patient’s side’. (No. 201, Master’s degree, >10 years’ experience, Private hospital/clinic) ‘Raise awareness of tinnitus to avoid common misconceptions about the condition – this often contributes to tinnitus distress’. (No. 229, Master’s degree, 10 years’ experience, Academic at a University)

Most reported that they had limited ability to assess or provide intervention services (*n* = 47, 53%). This was mainly because of limited knowledge and a lack of standard protocols or guidelines (*n* = 13, 15%) to assist them with this process. Other factors that limited the overall management of tinnitus included limited resources or funding (*n* = 28, 31.4%), language barriers (*n* = 30, 33.7%) and time constraints.

Most participants reported that further training in the overall management of tinnitus (*n* = 39, 39%), standard guidelines (*n* = 32, 32%), further research and continuous professional development workshops to support audiologists to effectively assist their patients would be beneficial.

## Discussion

This study revealed that participants lack confidence in the overall management of adult patients with tinnitus, citing limited preparedness, training, guidelines and resources as possible factors contributing to their limited service provision. With regard to training, capacity and preparedness, the results of the current study are similar to a local study conducted by Naidoo (2006). In that study, it was revealed that limited tinnitus services were provided because of limited training and its management was practised more routinely in the private than public health care sectors (Naidoo, 2006). Most participants also reported not attending specialised training for tinnitus, with only five participants attending courses that were facilitated in Denmark and the United Kingdom. Similar findings have also been observed in a study by Hoare et al. (2010), where 15% of audiologists specialised in tinnitus in the United Kingdom, with fewer clinicians undertaking training for counselling. In the current study, most participants had not undertaken any further specialised training and it is evident that extra courses for tinnitus are necessary for the overall management of tinnitus.

The feedback relating to the views and practices for the assessment of tinnitus indicated a variation in practices and a lack of standardised tinnitus practice among audiologists. In terms of intervention, participants also seem to lack confidence in this regard as their feedback bears similarity to their responses in relation to the assessment of tinnitus. These results are similar to a UK-based study in which it was noted that tinnitus practices are not standardised and this results in fragmented care with cost implications (Hoare et al., 2010). The serious impact of tinnitus and its lack of management could possibly lead to limited service provision (Holmes & Padgham, 2011).

In this study, most participants did not refer to any standard procedures for the assessment of tinnitus, while fewer participants sometimes followed standard procedures. The creation of guidelines specific to the needs of the community would serve as a guide to assist clinicians to manage tinnitus patients. Although there are currently no established guidelines in South Africa, it would be useful for audiologists to review international standards and make adaptations and recommendations as this would serve as a baseline for patient progress while empowering clinicians to create management plans for their patients. The majority of participants also responded positively for the development of tinnitus guidelines and also expressed an interest to develop a tinnitus management programme at their respective places of employment to assist with the overall management of tinnitus and offer services required by patients. Noise exposure, infections, and head and neck injuries are considered major risk factors of tinnitus that may require other health care professionals to intervene and assist patients where necessary (Baguley, McFerran, & Hall, 2013). In this study, it is evident that participants were familiar with the suggested risk factors associated with tinnitus and have also reported other conditions that included the intake of ototoxic medication for TB, HIV and AIDS and medication for depressed and anxious patients, in which tinnitus is likely to result as a side effect (Gülbay et al., 2006; Pillay-van Wyk et al., 2013). It is noted that participants made referrals mainly to ENT specialists, psychologists, dentists and neurologists. Referrals were also made to experienced audiologists with further training and who have a special interest in tinnitus. Regrettably, participants reported that referrals are not made to fellow health care professional as no staff are employed within the hospital or in closer surrounding areas. Collaboration with allied health care workers are essential to ensure that tinnitus patients are obtaining the appropriate medical services.

With regard to the assessment of tinnitus, participants included at least one of the options listed in the survey and these included case history, otoscopy and routine audiological testing. A lower response was attained for the inclusion of questionnaires. Similar results were obtained by Hoare et al. (2010) in which 91% of participants conducted audiological assessments with the inclusion of otoacoustic emission testing and acoustic reflexes as part of their assessment, with fewer participants conducting structured interviews. Audiologists also used unstructured interviews to determine the impact of tinnitus. The Tinnitus Handicap Inventory was the most commonly used questionnaire by participants, and the similar was noted in the United Kingdom in which the most prevalent measure of tinnitus severity was the use of questionnaires (67%) followed by the Tinnitus Handicap Inventory (45%) and the use of the Tinnitus Functional Index (TFI) at 18% (Hoare et al., 2010, 2015).

Participants conducted some form of tinnitus intervention by primarily providing counselling services, tinnitus retraining therapy and cognitive behavioural therapy. In a study by Hoare et al. (2010), similar feedback was obtained in which participants provided some form of management strategies that included hearing aids with directive counselling, sound generators, habituation therapies, stress management and psychological support, which included relaxation, acceptance and commitment therapy. There was no published data with regard to the most common practices used by audiologists to manage tinnitus patients and clinicians greatly relied on their clinical experiences (Naidoo, 2006). Tinnitus care is highly variable and the level of care differs among clinicians and countries (Hoare et al., 2015), and this is certainly noted in the current study.

The most significant challenge identified by participants is their inability to confidently assess and intervene adult patients with tinnitus. There seems to be much variation in tinnitus practice among audiologists in South Africa, with minimum to no standardisation for the overall management of tinnitus. Other international studies have also noted variations in tinnitus services between audiology departments among countries in the United Kingdom (Hoare et al., 2015). Most participants reported they do not refer to any guides in particular during the overall management of tinnitus and also displayed a lack of awareness or familiarity of resources that are used in other countries. This is important for the standardisation of practice, platform for sharing methods and success stories to assist those who may not be as confident when managing adults with tinnitus. Participants reported interest for a guide in place for the overall management of tinnitus as this would serve as a baseline for health care professionals. Although most participants provided some form of counselling to patients, they felt that their knowledge in this area is limited with fewer audiologists undertaking specialised training to counsel tinnitus patients. Participants reported it as being challenging to counsel patients after being seen by medical professionals who disregard tinnitus, indicating that nothing can be done about it (Moroe & Khoza-Shangase, 2014; Sourgen & Ross, 1998). It is necessary that this misconception changes towards a positive approach. This can only be achieved once audiologists become informed and begin to create awareness of the role to assist patients lead a better quality of life and help them effectively manage their symptoms (Hoare & Hall, 2010).

Regrettably, from the data it was identified that some institutions do not have any equipment or resources for the assessment of tinnitus. Some rural areas do not have ENT specialists employed and referrals for further medical review are not possible, placing patients at risk of not being treated for other life-threatening conditions that are associated with tinnitus. It is unfortunate that because of the invisible nature of tinnitus, resources are not often a priority and the sparse availability of resources in South Africa may affect the provision of tinnitus services (Moroe & Khoza-Shangase, 2014).

Participants reported that the lack of funding within the public health sector places a strain on the efficient functioning of the audiology departments. Because of budget constraints allocated for audiology, the main aim is to ensure that hearing aids are obtained as this is more crucial as opposed to purchasing expensive technology linked specifically to tinnitus maskers. It is imperative that audiologists continue to urge relevant levels of managements at their respective institutions to consider and support funding for much required resources, training and care.

Participants have noted that time constraints play a major role in the overall management of tinnitus. Because of a lack of staff in very busy hospitals, audiologists are limited in integrating much needed services into their clinical environments. The unequal distribution of audiologists in the public and private sectors has challenged the provision of audiological services to the greater population in South Africa (Swanepoel, 2006). Because of the fast pace nature of audiology clinics, longer time slots for tinnitus may work in the private health care sector (Schechter & Henry, 2002). Although English is a primary language in South Africa, isiZulu is the home language to 22.7% of the population (Statistics South Africa, 2011). From the data it was noted that 33.7% of participants have reported *language barriers* being an area of great concern. There are currently no tinnitus questionnaires translated into isiZulu and the language used in that particular context to suit the needs of patients whose first language is not English, thus impacting on the patient’s ability to understand and clearly express themselves through this process.

Not all patients view tinnitus as a medical condition, with feedback from some participants reporting tinnitus being a spiritual condition. In the unique multicultural society that we reside in, it is important to understand and respect the beliefs of patients as many South Africans prefer consulting with traditional healers along with modern health care medicine for health-related issues (Ross, 2008).

Participants have expressed the need for workshops to be held for both newly qualified clinicians and experienced audiologists who have an interest in the area of tinnitus. It was suggested that intensive training in the form of annual courses and educational workshops be implemented to keep updated regarding the current trends in tinnitus management. Participants requested to be trained on the relevant practical aspects of tinnitus, which include the assessment and management that would be useful within the clinical environment.

## Conclusions and recommendations

In South Africa, tinnitus services offered by audiologists seem to be limited. There are various contextual, linguistic and cultural factors that may influence the overall management of tinnitus in a clinical environment. A step forward would be to determine the measures taken by the clinician for the overall management of tinnitus and build on this to ensure that appropriate tinnitus services are being provided to patients. This may be partly accomplished through continual professional development workshops, adequate guidelines and resources for the overall assessment and intervention of tinnitus and advocacy with the aim of improving the quality of life of patients with tinnitus.

The teaching curriculum at universities should be reviewed so that students confidently enter the workforce being more prepared and knowledgeable on how to assess and manage patients with tinnitus. Feedback from participants recommended that more clinical case studies be included, and to ensure familiarity of tinnitus the course content should be provided earlier in the degree. According to Hoare et al. (2010), participants reported that patient empowerment and a positive attitude towards tinnitus make up a successful tinnitus management plan. From the current study, it is evident that audiologists have acknowledged the gaps in their professional capacity to assist tinnitus patients and indicated their need to be trained further. Participants also accepted that being informed would enable them to confidently manage patients with tinnitus. The majority of participants strongly recommended the need for a guide or protocol to aid audiologists with the overall management of tinnitus patients. It was emphasised that these guides should be developed specifically to suit the context of South African patients in which cultural and linguistic aspects should be considered. It is recommended that experienced audiologists collaborate to create a task force dedicated to tinnitus. Although it would be extremely beneficial for a guide to be in place, it is important that audiologists are mindful that the overall management of each tinnitus patient may differ.

Some participants felt that there is a lack of awareness among health care professionals regarding the role of audiologists towards patients with tinnitus and it is vital that awareness is created to efficiently assist patients. Furthermore, a lack of research or information regarding tinnitus and its overall management within the South African context was reported. As a result, the creation of a local website dedicated to tinnitus was recommended by participants in which an online forum would serve as a platform for audiologists, where health care professionals may share knowledge and resources that are easily accessible. Consideration to develop a website to support patients would also be useful for those who do not have direct access to tinnitus services at their hospitals and this would also be advantageous to patients within the South African context as some patients may easily access Wi-Fi.

### Research implications and limitations

Future research on a larger scale should be considered and incorporating a mixed-method design over a longer period with the aim of creating evidence-based practices that is relevant to the needs of patients with tinnitus in South Africa. This could additionally consider the cultural and linguistic factors when managing tinnitus patients. Some patients do not consider tinnitus as a medical condition, but rather a result of spiritual causes. More research in this area can be used to advise practitioners accordingly. The results obtained were based on a small sample size and may lack generalisability. Results should, therefore, be interpreted with caution.
